# Appraising the decision-making process concerning COVID-19 policy in postsecondary education in Canada: A critical scoping review protocol

**DOI:** 10.3934/publichealth.2023059

**Published:** 2023-11-13

**Authors:** Claudia Chaufan, Laurie Manwell, Benjamin Gabbay, Camila Heredia, Charlotte Daniels

**Affiliations:** 1 School of Health Policy and Management, York University, 4700 Keele St, Toronto, ON, M3J 1P3, Canada; 2 Wilfrid Laurier University, Canada; 3 Toronto Metropolitan University, Canada; 4 School of Health Policy and Management, York University, Canada

**Keywords:** COVID-19, critical policy analysis, decision-making, governance, academia, postsecondary education, medical mandates

## Abstract

**Background:**

Responses to COVID-19 in Canadian postsecondary education have overhauled usual norms and practices, with policies of unclear rationale implemented under the pressure of a reported public health emergency.

**Objective:**

To critically appraise the decision-making process informing COVID-19 policy in the postsecondary education sector.

**Methods:**

Our scoping review will draw from macro and micro theories of public policy, specifically the critical tradition exemplified by Carol Bacchi's approach “What is the problem represented to be” and will be guided by Arksey and O'Malley's framework for scoping reviews and the team-based approach of Levan and colleagues. Data will include diverse and publicly available documents to capture multiple stakeholders' perspectives on the phenomenon of interest and will be retrieved from university newsletters and legal websites using combinations of search terms adapted to specific data types. Two reviewers will independently screen, chart, analyze and synthesize the data. Disagreements will be resolved through full team discussion.

**Discussion:**

Despite the unprecedented nature of the mass medical mandates implemented in the postsecondary sector and their dramatic impact on millions of lives—students, faculty, staff and their families, friends and communities—the decision-making process leading to them has not been documented or appraised. By identifying, summarizing and appraising the evidence, our review should inform practices that can contribute to effective and equitable public health policies in postsecondary institutions moving forward.

## 1. Introduction

Universities and colleges (hereafter “postsecondary institutions”/academia) across the world have adopted a range of policy responses to manage the COVID-19 crisis, leading to an overhaul and reinterpretation of usual norms and practices in postsecondary institutions. While national, provincial and local public health regulations have set the frame within which these responses were formulated, developed and implemented, actual policies have differed among postsecondary institutions across the world, within the same countries and even among localities. A unifying theme, however, has been that policies were developed and implemented under the pressure of a reported public health emergency. As a result, the rationale has not always been clear, nor has the process always been transparent. In Canada, for example, medical mandates were implemented, seemingly in accordance with public health regulations/recommendations [1]–[5]. However, these mandates often remained in place into the winter of 2023 [6],[7], well after these regulations had been lifted and the recommendations were no longer in effect [8],[9]. At the time of this writing, and even months after the WHO determined that COVID-19 is no longer a “public health emergency of international concern” [10], 101 postsecondary institutions across 19 US states—including Harvard University, Johns Hopkins University and Rutgers University—plan to maintain vaccination mandates as a registration requirement for the fall of 2023 [11]. Nevertheless, despite the unprecedented nature of these policies and their dramatic impact on the lives of millions—students, staff, faculty and their families—little is known about the decision-making processes leading to them; and the power dynamics that, as scholars have argued, shape policy-making [12], have been insufficiently explored.

## 2. Background

### 2.1. Debates around the medical-scientific evidence informing mass mandates

Appraising the decision-making process leading to COVID-19 medical mandates in the post-secondary sector is especially pressing in light of mounting evidence questioning their wisdom. On the one hand, what has been deemed the “scientific consensus” on COVID-19 remains largely unchallenged. In October 2020, an article entitled “Scientific consensus on the COVID-19 pandemic: We need to act now” announced in *The Lancet* that COVID-19 had a lethality “several-fold higher than the seasonal flu”, that the healthy and young could still experience high risk of poor outcomes, that there was little reason to rely on natural immunity and that there was a pressing need for mass masking, lockdowns, rapid testing, contact tracing and isolation to control viral spread and transmission, with anything less being a “dangerous fallacy unsupported by scientific evidence” [13] (p. e71). The call to “act urgently” to counter challenges to this consensus was echoed by a new field of academic inquiry, “misinformation studies” [14], whose examination reveals an intriguing interplay between the scientific enterprise and party politics [15]. At the time of this writing, the World Health Organization, which spearheaded this consensus, continues to assert that “everyone, everywhere, should have access to COVID-19 vaccines” and that mass vaccination is instrumental to assuring the well-being of global populations [16].

On the other hand, there is little disagreement in the medical and public health communities that when formulating policy, not only (actual or anticipated) benefits but also (actual or anticipated) risks *for the recipient population* should be considered, for prudential and bioethical reasons [17]. Take, for instance, vaccine mandates. Already in the summer of 2021, it became clear that mass vaccination did not correlate with lower cases of COVID-19. A study published at the time in the *European Journal of Epidemiology*, of 68 countries and 2,947 US counties, showed no correlation between COVID-19 vaccination rates and cases [18]. This feature of COVID-19 vaccines could have been foreseen in the original trials, which did not include transmission, hospitalizations or deaths as clinical endpoints [19]. There was already also solid evidence that a positive PCR test—intended “for research [and not] diagnostic procedures” [20]—was not equivalent to clinical illness, calling into question the policy of testing as a means to determine the magnitude of a perceived health threat. It also became clear that there was little evidence to support mass vaccinating healthy people when the largest study ever conducted, of close to 10 million individuals in Wuhan, China, revealed no positive tests amongst 1174 close contacts of asymptomatic cases [21]. At the present time, more than three years into the onset of COVID-19, it is well-established that children, adolescents and young adults are at much lower risk of severe disease, hospitalization and death than older adults [22],[23] and that persons with comorbidities are at significantly higher risk than those without them [24],[25] (We assume official statistics on COVID-19 health outcomes at face value. We note however that these statistics are highly questionable, given the ambiguity, acknowledged by major public health agencies, between, for instance, deaths “linked” to COVID and deaths “because of” it. Already in 2021, the CDC admitted that about 95% of “COVID deaths” were “linked” to at least 4 co-morbidities [24] that, in pre-COVID times, would have been recorded as causes of death. Similarly, when on March 11, 2022, Public Health Ontario announced that it was revising its definition of “COVID-19 hospitalizations and deaths,” 54 percent of the first were removed because they had been “admitted for other reasons but have since tested positive for the virus,” whereas deaths were reduced by over 400 [26]. Muddied operationalizations of critical indicators could ultimately mean that counts of hospitalizations, disease and deaths have been grossly overestimated.). International evidence has also indicated that the risk of outbreaks in educational institutions, where the population is mostly young, was very low [27], and data from North American public health agencies only list acute care, congregate living, correctional and long-term care facilities—all settings with very high vaccination rates—as accounting for 100% of their own recorded “outbreaks” [28], excluding educational institutions entirely.

There is also little disagreement that under conditions of uncertainty about the best options, the precautionary principle should guide policy, especially when it impacts millions. This principle involves “shifting the burden of proof to the *proponents* of an activity; exploring a wide range of alternatives to possibly harmful actions; and increasing public participation in decision making” [29] (p. 871). As applied to COVID-19 inoculations, the burden of proof should lay on its proponents, alternatives to harmful effects should be considered, and public participation should be guaranteed, especially when safe alternatives do exist, such as early treatment with repurposed drugs with excellent safety profiles [30]–[33], when claims that COVID-19 vaccines stop viral spread have long been abandoned, even by leading public health agencies [34], when evidence that boosting with updated (i.e., bivalent or other) COVID-19 inoculations correlates with a *higher* risk of infection is mounting [35], and when the risk-benefit ratio of mass inoculation mandates for a largely young and healthy population does not favor this approach [36]—In fact, a growing number of adverse events post COVID-19 vaccination have been documented, including severe disability and death [37]–[41].

### 2.2. The corporatization of postsecondary education

Be that as it may, the increasing corporatization/privatization of postsecondary education, along with its influence on the policy process within the sector, is well documented and has rightfully concerned education scholars, who have argued that whatever the normative goals, private interests—from the food, the banking, the military and the medical industries—are increasingly overriding the interests of the public [42],[43]. Still, postsecondary institutions in Canada remain largely public, i.e., significantly reliant on public funding, i.e., government funding [44], and, as public corporations, are expected to abide by the five core principles of “good governance”: 1) *accountability*, i.e., “the process whereby organizations and the individuals within them take responsibility for their decisions and actions”; 2) *leadership*, i.e., “setting the ‘tone at the top’ [if an] organization is to embrace good governance”; 3) *integrity*, i.e., “acting in a way that is impartial, ethical, and not missing information or resources”; 4) *stewardship*, i.e., “looking after resources on behalf of the public [by] maintaining or improving capacity to serve the public interest”; and 5) *transparency*, i.e., a quality “achieved when decisions and actions are open [and] stakeholders, the public, and employees have access to full, accurate, and clear information” [45] (p. 5–6). Corporatization aside, if postsecondary institutions are to remain trustworthy and live up to these five principles, they owe the public a comprehensive and transparent account of the decision-making process leading to policies—COVID-related or not—in their midst.

### 2.3. Drivers of the policy-making process in postsecondary education

A cursory examination of the academic literature reveals that policymaking in postsecondary education is anything but transparent and rational and that, claims about “evidence-based” policymaking notwithstanding, outcomes are serendipitous, significantly shaped by power relations, influenced by multiple micro- and macro-level factors, historically and geographically contingent and hard to predict. For instance, Enders has argued that policy analysis in postsecondary education has tended to concentrate on the *effects* of policy, ignoring the “input” aspect, meaning the forces resulting from the increasing influence of international relations [46]. While Enders's work refers to the impact on educational policy of European governance bodies (e.g., European Commission, European Central Bank) that limit sovereignty in domestic policy, it can well apply to Canada, whose domestic policy space is similarly constrained by international forces: for instance, trade agreements that already some thirty years ago were predicted to influence Canadian postsecondary education policy [47]. In turn, Axelrod has discussed federal Liberal party policymaking in postsecondary education between 1990 and 2000, proposing that the “unprecedented initiatives” undertaken by the Liberal Government of Prime Minister Jean Chretien reveal that policymaking in postsecondary institutions is influenced more by “a complex and shifting interplay of ideas, interests, [...] networks and lobbyists” than by objective criteria and neutral considerations [48] (p. 143).

Concerning COVID-19 policy, a preprint article by Brubacher et al. [49] has argued that in British Columbia, the *appearance* of the prevalence of evidence-based policy notwithstanding, interviews with key informants—BC elected officials, provincial and regional-level health officials, and civil society actors—revealed “lack of clarity” concerning “what evidence was used,” uncertainty about the ability of public health actors to independently “articulate evidence to inform pandemic governance,” “lack of coordination and continuity across data sources, and a lack of explicit guidelines on evidence-use in the decision-making process, which resulted in a sense of fragmentation”—in short, important and multiple barriers to evidence-based policy [49] (p.2)—with “evidence” meaning evidence for the medical/scientific grounds informing public health policy. A similar situation appears to obtain concerning COVID-19 policymaking in the education sector. For instance, in their analysis of the reopening plans of over 10,000 US school districts, Hartney and Finger concluded that “contrary to the conventional understanding of school districts as [...] non-partisan actors [it was] politics, far more than science [that] shaped school district decision-making” and that “teacher union strength best explains how school boards approached reopening” [50] (p.1). Similarly, concerning postsecondary education, Felson and Adamczyk applied multilevel modelling to a sample comprising 87 percent of US nonspecialized two- and four-year public and private colleges, concluding that reopening decisions as of October 2020 were driven more by budgetary and political considerations than by university leaders' *perceptions* of public health risks (emphasis added) [51]. Similarly, in their analysis of 451 postsecondary US institutions, Johnson and Roberto concluded that “political forces may influence university administrators' decisions about” reopening the fall 2020 semester, countering administrative theory that “prescribes good governance as premised on political neutrality” [52] (p. 271). Most studies however are quantitative and do not include documentary material, nor have they engaged the Canadian context.

### 2.4. The relevance of a critical lens in policy analysis

A decades-long tradition of scholarship has underscored the importance of appraising the power dynamics in public policy making [12],[53], and many scholars in the field have proposed to bring a critical lens—in policy matters and beyond—to comprehensive and systematic reviews of the literature [54],[55], including scoping reviews [56],[57]. While what is meant by “critical” differs among scholars, overall they agree that such approaches should probe the assumptions underlying knowledge claims and, in the case of reviews, these should go beyond what is all too often a mere “listing or catalogues of previous research” to consider “if [authors'] conclusions can be justified by the evidence” [55] (p.159). An exemplar of the critical tradition as applied to policy studies is the work of Carol Bacchi and her approach to policy analysis, “What is the problem represented to be?” (WPR) [53],[58]. This approach examines the process whereby societal issues amenable to policy interventions—in this case, the challenge of managing COVID-19 within postsecondary institutions—become framed as problems in the first place. While mainstream approaches envision policy analysis as a technical task, take at face value dominant representations of policy problems, and seek solutions within the boundaries of these representations, for researchers applying the WPR approach, problem definition and framing are interpretive endeavours, influenced by power relations. Applied to our case, our critical scoping review will examine how decision-making concerning COVID-19 policy has been influenced by competing perspectives on what counts as reliable scientific evidence, by the power/class structure within universities (e.g., management versus faculty; tenured versus contract faculty; faculty versus students; administrative staff vs. faculty and students; individuals vs. collectives); and by potential conflicts of interests (e.g., funding streams prioritizing certain curricular or research areas over others). Our “informants” will be documents that we will treat as “social facts,” i.e., organized to reflect actual power relations and convey meanings [59] (p.27).

A preliminary search of JBI and the Cochrane Database for Systematic Reviews revealed no current or underway systematic or scoping review on our phenomenon of interest. Therefore, our objective is to fill this gap by identifying evidence for, and appraising, the decision-making process concerning COVID-19 policy in Canadian postsecondary institutions, thus contributing to the “good governance” principles of accountability and transparency, both critical to the public's trust in societal institutions and to the legitimacy and democratic nature of the process. By identifying, summarizing and appraising relevant data, our critical scoping review will inform best practice guidelines for institutional leadership and the development of effective and equitable public health policies in postsecondary institutions moving forward. Because all data is publicly available, ethics approval and consent to participate are not applicable.

## 3. Material and methods

### 3.1. Protocol design

Traditional systematic or scoping reviews generally evaluate “interventions.” In contrast, other review types, like ours, seek to understand “diverse information needs [of] policymakers [and instead] focus on analysing human experiences and cultural or social phenomenon,” labelled “phenomena of interest” [60] (p. 2). Our “phenomenon of interest” is the decision-making process leading to COVID-19 policy responses in Canadian postsecondary institutions, with an emphasis on selected pharmaceutical (e.g., vaccine mandates) and nonpharmaceutical (e.g., campus closures) interventions. This protocol has been registered with the Open Science Framework (https://osf.io/pbthn). The review will follow the Preferred Reporting Items for Systematic Reviews and Meta-Analyses extension for Scoping Reviews (PRISMA-ScR) Checklist and will document amendments to the protocol [61].

### 3.2. Objectives of the review and review questions

The purpose of our scoping review is to critically appraise the evidence informing the decision-making process concerning specific health-related COVID-19 policies-mask/vaccine mandates and campus closures-in Canadian postsecondary institutions. The following review questions, the first and the last drawn from the WPR approach [62], will guide our inquiry:

- What were the “problems” represented to be in COVID-19 policy responses in postsecondary education in Canada? What presuppositions underpinned these representations and how did they come about? (hereafter *PROBLEM REPRESENTATION*).
- What bodies or individuals were responsible for making decisions? (hereafter *DECISION-MAKING*).
- What processes of consultation, if any, were carried out, to arrive at a given decision? How were members of the academic community (e.g., students, faculty, staff) included (or not) in the decision-making process? (hereafter *DEMOCRATIC GOVERNANCE*).
- What scientific (i.e., medical and public health) evidence informed the decision-making process? (e.g., guidelines from public health agencies; medical experts, other?) (hereafter *EVIDENCE*).
- How did institutional policies align (in content and chronology) with policies implemented by public health authorities? (hereafter *CHRONOLOGY*).
- What equity, diversity, inclusivity and bioethical (e.g., informed consent) criteria were considered in the decision-making process? What alternatives (i.e., accommodations) were offered to individuals unable or unwilling to comply with official policy for medical, religious or personal (matters of conscience) reasons? (hereafter *ETHICAL CONSIDERATIONS*).
- What was the role of funding (e.g., monies received from government or private parties) in policy formulation/development/implementation? (*CONFLICTS OF INTEREST*).
- What was left unproblematic in the representation of “policy problems” addressed by academic policies? Could they have been conceived differently? Who was affected by these representations? (hereafter *MISSED ALTERNATIVES*).

### 3.3. Data identification

#### 3.3.1. Types of data

Decision-making in academic institutions occurs at various levels, and details about the process are often documented in the grey literature. Grey literature typically covers work that is not published or distributed through commercial or academic organizations yet still contains important research, academic, technical and other information such as that found in institutional reports, theses, conference proceedings, preprints, patents, presentations, field notes, academic courseware, lecture notes, evaluations and many more. In this review, the grey literature relevant to decision-making related to COVID-19 policy in academia will be sourced primarily from 1) documents containing information on legal decisions affecting Canadian postsecondary institutions (hereafter *Legal Documents*); 2) publicly available communications from faculty, staff, and student associations (hereafter *Association Documents*); 3) articles in the academic magazine *Academic Matters* (hereafter *Magazine Documents*); 4) publicly available meeting agendas, minutes, reports and motion records of meetings of decision-making academic bodies (hereafter *Governance Documents*); 5) material from the public news and announcements (blog) section of university websites (hereafter *News Documents*).

#### 3.3.2. Sources for data retrieval

The databases for retrieving the five types of documents will include 1) the *Canadian Legal Information Institute* (CanLII), a non-profit database of legal documents with information on legal decisions affecting Canadian postsecondary institutions; 2) the main websites of faculty, staff, and student associations (e.g., website of the *University of Toronto Faculty Association*); 3) the website of *Academic Matters*; 4) the websites of five Canadian universities, selected due to a combination of reasons-diverse geographical location, recognized influence in the health sciences, prominence of their academics in the Canadian news media during the COVID-19 crisis, or the fact of being an outlier within provincial policy norms.

#### 3.3.3. Search methods for identifying data

Documents will be collected spanning about 3 years, from January 2020 through August 2023. For the *Legal*, *Association* and *Magazine* data sets, only documents that answer the question “*What is known from the existing literature about the decision-making process in Canadian academic institutions concerning COVID-19 policy?*” will be included for detailed analysis. Combinations of the following search terms, adapted to the particularities of each data set, will be used to identify these documents: COVID AND policy AND [higher education OR postsecondary OR academia OR university] AND / OR decision-making. Records of searches will be created for the following, as needed: Total Records Identified; Duplicates Removed; Records Screened (Titles Assessed); Records Eligible (Abstracts Read); Records Included (Downloaded); Records Assessed; (Summarized). Two researchers will independently review the collected documents and select those to include based on the following additional inclusion criteria, with at least 80% agreement [63]: must be related to non-pharmaceutical interventions (NPIs) (i.e., institutional closures, remote work, physical distancing, hygiene, and masking) or pharmaceutical interventions (i.e., vaccination). Additional exclusion criteria will be applied including the following: not in English or not in Canada.

Relevant documents from the *Governance* and *News Documents* sets will be identified through a multistep process that involves automatically scoping the source databases (see 3.2.1) with tailor-made Python-based web crawlers that capture all documents and/or webpages containing certain key substrings (“covid,” “sars-cov,” “coronavirus,” and “pandemic”) intending to capture any and all references made to the COVID-19 pandemic and its response, and manually filtering the captured documents to identify those that discuss the areas in which the majority of Canadian postsecondary institutions developed/enforced COVID-19 policies (e.g., social distancing and, by extension, capacity limitations, campus closures and remote learning; masking; and proof-of-vaccination policies); and/or discuss the messaging of COVID-19 policies and policy decisions by university authorities; and/or discuss the decision-making process of COVID-19-related policies (e.g., the delegation of power to create, influence); and/or discuss collaboration with government bodies, NGOs and/or other institutions in the context of forming said policies. The use of Python-based web-crawlers, in combination with a manual filtering process, is required for these two data sets because not all the source databases have reliable, built-in search functionalities, and the filtering criteria are complex. The full Python web-crawler scripts employed to search each database, including their master template and numbers, will be provided in the completed scoping review as an appendix.

#### 3.3.4. Data selection

For all data sets, before including documents for assessment, one reviewer will conduct a preliminary search for each dataset to discard irrelevant material and narrow retrieved documents to a number manageable considering our human resources and timeline. Upon this initial screening, two reviewers will independently screen the remaining documents in relation to our research questions and eliminate those that do not meet the inclusion criteria. Disagreements will be resolved by full team discussion. Excluded documents with relevant contextual material may be retained and narratively summarized in the background section of the completed review.

### 3.4. Data analysis and synthesis

#### 3.4.1. Data charting

Data charting will include features of documents (e.g., type/author/date), intended audience (e.g., university community vs. community at large), social actors participating in a decision (e.g., management vs. faculty vs. staff vs. students), policy area under discussion (e.g., campus closure; vaccination mandate), evidence informing the decision (e.g., public health agencies; original medical research) and factors potentially influencing a given decision (e.g., funding streams), as well as “problem representation” and “missed alternatives,” as described in section 2.2. Before beginning full charting, two researchers will independently chart data from a common sample of documents, and the team will meet to calibrate the approach and discuss the results. Tables for all included documents will be created and included as appendices in the final review. The charting process will be assisted by *Dedoose*, a cloud-based application that helps to organize and analyze a wide range of data. We will monitor interrater reliability on a regular basis during the charting stage and act if it falls below 80% [63].

#### 3.4.2. Data synthesis

We will use qualitative thematic synthesis to transform the data into themes [64],[65], applying a hybrid, deductive/inductive approach that involves reading and rereading the evidence to identify themes, comparing the themes as the analysis progresses and meeting regularly to resolve uncertainties or ambiguities. Our guiding review questions have helped to identify preliminary themes, but as we assign data to them, we will assess if they are supported by the data or require revision or addition of new themes [66]. We will report both qualitative thematic synthesis and frequency distributions to illustrate the strength of support for themes [67].

#### 3.4.3. Subgroup analysis

We will perform subgroup analyses to compare findings according to 1) type of document, 2) intended audience, 3) social actors issuing a document/participating in a decision, 4) policy area under discussion, 5) evidence informing a decision 6) and factors potentially influencing a given decision, as described earlier.

#### 3.4.4. Quality assessment and risk of bias

The scoping review approach specifically excludes the need to assess the risk of bias [68]. Especially in our case, we will not include peer-reviewed articles—whose quality and risk of bias we may otherwise have chosen to assess—as data but rather incorporate them as background when relevant. Furthermore, our data will be “biased” by its very nature, meaning they will likely favour one position over another (e.g., magazine articles that inform *and* attempt to persuade audiences). In fact, our objective is precisely to appraise if and to what extent the decision-making process concerning COVID-19 policy was informed by evidence-based sources and normative standards in bioethics and equitable policy, so assessing for quality and bias and excluding documents that failed to meet normative standards would defeat this objective. Finally, and in contrast to scoping review authors in the field of education appraising the peer-reviewed literature [69], the diverse nature of our data precludes us from applying traditional instruments to assess quality.

## 4. Discussion

To the best of our knowledge, there is a dearth of scholarly analysis concerning the decision-making process around COVID-19 policy in Canadian postsecondary institutions, despite the unprecedented scope and nature of these policies in these institutions' history, the contested nature of the “official” medical and public health positions and their dramatic impact on the lives of millions of Canadians—students, staff, faculty, their families and the broader community. As we have argued, research on this phenomenon has largely focused on assuming as a “given” the policies that were implemented or assessing whether they “succeeded/failed” according to the assumptions and standards set by the social agents—individuals and organizations—implementing those policies, without appraising the decision-making process per se and the evidence informing this process, a gap that our scoping review will fill. By identifying, summarizing, and critically appraising both process and evidence, we aim to inform practices that can contribute to effective and equitable public health policies in postsecondary institutions moving forward.

Still, we expect practical challenges: For instance, it may be difficult to select from within the large number of documents identifiable through specific websites. Further, we may also encounter gaps in the decision-making process that may require access to non-public records through Access to Information Act requests. Conversely, the strengths of our team include the fact that all researchers have lived experience of the phenomenon of interest as members of academic communities—as tenured/contract faculty, research staff and former/current students—and are trained in multiple and relevant academic/professional disciplines—e.g., critical policy studies, biomedical/psychological sciences and qualitative/quantitative methodologies. Therefore, and given our choice of a team-based, critical scoping review approach, we are confident that we can address these challenges collaboratively, drawing from this collective experience/expertise. We also intend to disseminate our findings through professional conferences, refereed publications, policy briefs and meetings with members of the Academy to discuss lessons learned and knowledge translation strategies.

## 5. Preliminary timeframe

Upon protocol registration, an eight-month timeframe will be dedicated to data gathering, selection, charting, and manuscript drafting with a tentative completion date of April 2024.

## 6. Plans for updating this review

Claudia Chaufan will be responsible for updating the review within the proposed timeframe.

## Author contributions

C. Chaufan designed the scoping review, wrote this protocol and will oversee all steps of the project. LM, BG, CH and ChD contributed to writing this protocol and will assist with drafting the final review. BG will assemble the Python scripts for scoping university databases as necessary. CH will provide methodological expertise. All team members will participate in data selection, extraction and synthesis, and have read and approved this protocol.

## Use of AI tools declaration

The authors declare they have not used artificial intelligence (AI) tools in the creation of this article.
